# The Role of the Flipped Classroom Method in Short-Term and Long-Term Retention Among Undergraduate Medical Students of Anatomy

**DOI:** 10.7759/cureus.45021

**Published:** 2023-09-11

**Authors:** Payal Kasat, Vishwajit Deshmukh, Gayatri Muthiyan, Gugapriya T. S., Bharat Sontakke, Smita R Sorte, Aditya M Tarnekar

**Affiliations:** 1 Anatomy, Dr. B. C. Roy Multispeciality Medical Research Center, Nagpur, IND; 2 Anatomy, All India Institute of Medical Sciences, Nagpur, Nagpur, IND; 3 Physiology, All India Institute of Medical Sciences, Nagpur, Nagpur, IND

**Keywords:** long-term retention, short-term retention, traditional module, flipped module, teaching learning method

## Abstract

Introduction

Medical education is changing towards more flexible, effective, active, and student-centred teaching strategies that reduce the limitations of traditional methods of education. Recently, the flipped classroom method has been suggested to support this transition. However, research on the use of flipped classroom methods in medical education pertaining to short- and long-term retention of the topics is at an early stage. The present paper aims to determine and compare the effects of traditional and flipped classroom methods on first-year medical students' short-term and long-term retention.

Method

Fifty first-year medical students were subjected to traditional and flipped classroom modules in the form of five sessions each on gross anatomy topics of the thoracic region. These sessions were conducted during independent teaching slots for anatomy. Assessments were done at the completion of each module for both methods. Then, after a gap of two months, the students were again assessed on the content taught in the modules as a part of formative assessment. The data so obtained were compared and analysed statistically. Ethical approval was obtained prior to beginning the study. Written informed consent was obtained from the participating students.

Result

A total of 50 first-year medical students participated in the study. 33 (67%) participants were males, with a median age of 19.47 years, and 17 (33%) participants were females, with a median age of 19.39 years. The assessment scores showed differences between the two methods of teaching in the short and long term. The flipped classroom method was observed to have significant short-term retention with a p-value <0.0001, which is statistically significant.

Conclusion

The study concludes that the flipped classroom method serves as an advantageous tool and motivating factor for effective learning, understanding, and retention of conceptual and factual anatomical content.

## Introduction

In historical times, teaching was in the form of "sage on the stage," where a professor imparted knowledge by lecturing to the class [1]. The traditional classroom portrays teachers as content providers and students as receivers. Thus, in traditional classroom settings, students have been made passive in the process of knowledge acquisition [1]. In contrast to traditional methods of teaching anatomy, flipped classroom teaching is a pedagogical intervention that is beneficial for students. It is a type of blended teaching-learning method in which students are introduced to the content to be learned at home in the form of presentations, pictures, videos, notes, etc., and are expected to discuss these concepts and apply the content during the flipped sessions [2].

Thus, the content of didactic lectures, which usually take place during face-to-face time, is prerecorded and made available for students before the flipped classroom sessions. During flipped classes, they perform learning activities such as exercises, projects, or discussions. Thus, the students can gain knowledge and concepts in order to attain higher levels of Bloom’s taxonomy [3,4]. A flipped classroom inverts the typical cycle of content acquisition and application. Class time and conventional homework time are exchanged. Homework is done first, and class time is effectively used for personalised learning, which serves as a new paradigm in medical education [3,4].

Recent studies of learners perceptions of the flipped classroom among health professionals found an overwhelmingly positive response from students after attending the flipped classroom. Students also highly appreciated the regular formation of small groups for discussion-based activities, which are face-to-face and help to change their attitude towards learning, furnish motivation to learn, increase their level of engagement, and most importantly, increase interest in the subject. Students expressed high levels of satisfaction with pre-class videos and presentations because they have unrestricted access to pre-recorded video lectures at any time and place, at their convenience, and at their own pace [5,6].

The purpose of this research was to determine and compare the effects of the flipped classroom method and the traditional method on first-year medical students' short-term and long-term retention.

## Materials and methods

The prospective, mixed-methods study was conducted on first-year medical students. Prior ethical approval for the study was obtained from the Institutional Ethics Committee (IEC). Written informed consent was obtained from the students who were willing to participate in the study. A total of 50 first-year medical students participated in the study.

**Figure 1:**
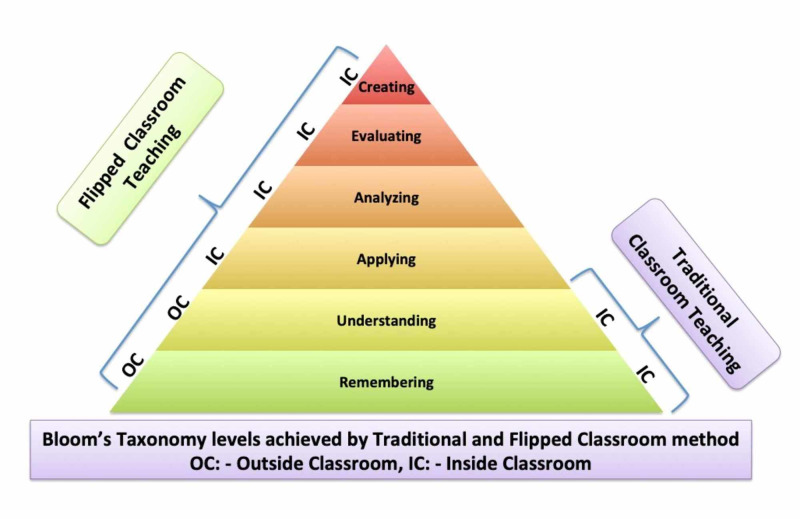
Illustration showing Bloom’s taxonomy levels achieved by Traditional and Flipped classroom method.

The study was conducted in the Department of Anatomy of an undergraduate medical teaching institute. All 50 students were subjected to traditional and flipped modules on different occasions. Gross anatomy topics from the thoracic region were divided into five traditional and five flipped module sessions. The topics included were appropriate for the level of a first-year medical student. These sessions were conducted during independent teaching slots for anatomy. Full attendance was ensured for these sessions by encouraging the students to participate in the study (Figure 1).

For the traditional module, routine large-group didactic sessions were conducted. Students attended five lecture sessions of one to two hours (attendance was recorded). The pace of delivery was determined by the lecturer. Case-based scenarios were presented by the teaching faculties for each topic. Any questions volunteered by students during the hour were answered. These were followed by short-term assessments. Students were provided with PowerPoint presentations, practice questions, and diagrams after each session.

In the flipped module, students were provided with pre-recorded videos prepared by the anatomy faculties, PowerPoint presentations, PDF documents from reference textbooks, etc. one week prior to the scheduled sessions on the WhatsApp group. They were instructed to: (i) watch the prerecorded videos; (ii) read the study material (textbook, notes, or PPTs); (iii) make mind maps or notes for the topic; (iv) practice questions that are given for the topic; and (v) jot down queries that arise during the pre-flipped session.

After this, during the flipped classroom sessions, the students were divided into small groups of five. For each group, a facilitator was assigned. In the beginning, the facilitator would provide case-based scenarios for engaging students in higher levels of critical thinking and problem-solving. The facilitator will observe the discussion passively and will intervene only at the end to give feedback, clarify misconceptions, help understand challenging concepts, resolve doubts, and conclude the session. In the end, the facilitator would give personalised assignments to the students.

During these discussions, the students were expected to engage in: (i) peer-teaching by systematically presenting the topic; (ii) cooperative learning by answering the queries of colleagues based on their understanding; and (iii) customised learning by seeking explanations from the facilitator for any queries left unsolved.

Short-term assessments were done at the end of each module for both methods. The students underwent routine dissection sessions after short-term assessments for both methods. Then, after a gap of two months, the students were assessed on the content taught in the modules as a part of formative assessment. Extended multiple-choice questions of higher order were utilised for the assessments. Additional theoretical questions included in the formative assessment were based on clinical vignettes. Equal weightage was awarded to the topics from both methods during the formative assessment.

Feedback from students about both methods was obtained. The results were recorded and tabulated. The different parameters recorded were: (i) short-term assessment scores immediately after completion of the traditional and flipped teaching modules; and (ii) long-term assessment scores two months after completion of the traditional and flipped teaching modules.

The data obtained were statistically analysed and compared using GraphPad Prism 10 software. The mean, range, and standard deviation were calculated. A student t-test with a two-tailed distribution was applied for the comparison of values of different parameters. The p-value of <0.05 was considered statistically significant.

In the Indian context, the eligibility criterion for appearing in professional examinations is a 35% score in the formative examinations. A minimum of 50% scores in professional examinations is essential for clearing these examinations. Hence, we have compared the short-term and long-term assessment scores of students with reference to their short-term assessment scores, or >50% and 35%, to determine the improvement in their performance.

## Results

**Figure 2:**
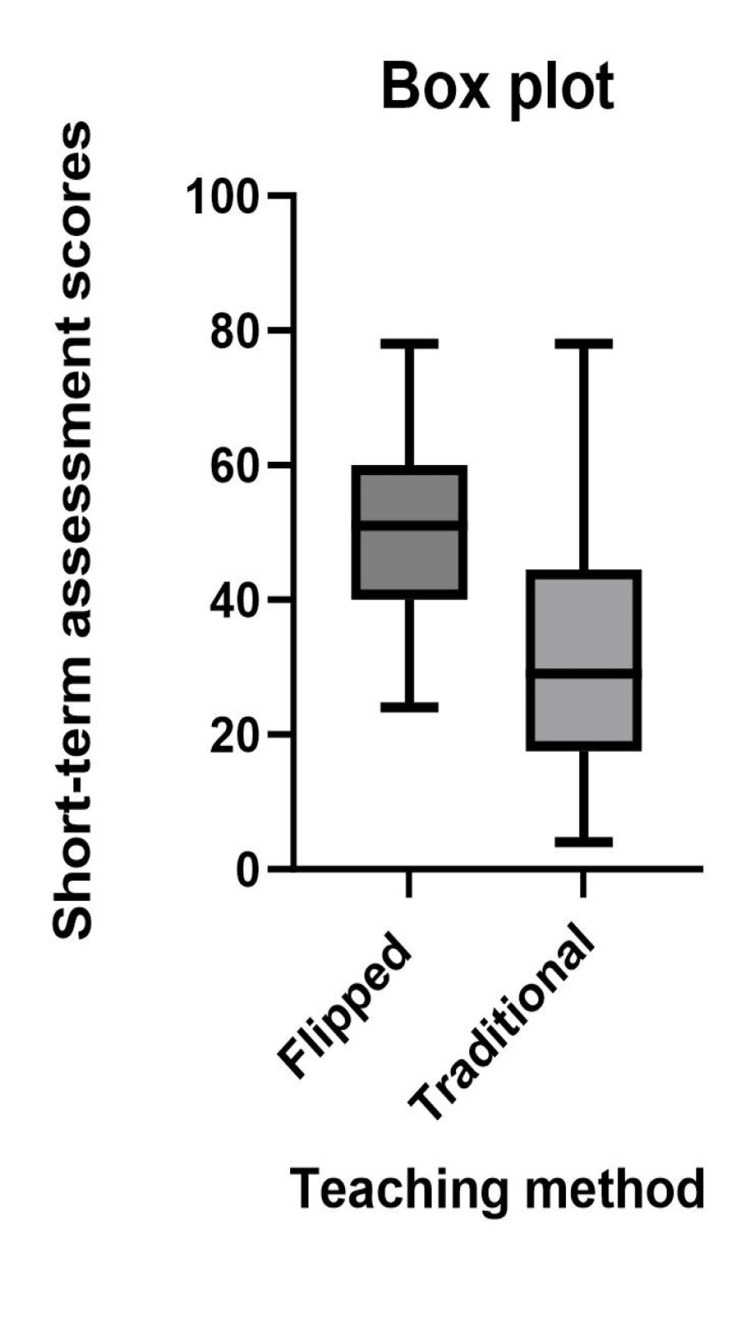
Comparison of short-term assessment scores between flipped and traditional modules.

Out of 50 first-year medical students, 33 (67%) participants were males, with a median age of 19.47 years, and 17 (33%) participants were females, with a median age of 19.39 years. The difference between the assessment scores for the short-term between the flipped module and the traditional module was statistically significant with a p-value of <0.0001 (Table 1 and Figure 2).

**Table 1:** Comparison of short-term scores and long-term scores between traditional and flipped modules.

Assessment		Name of module		p-value
		Traditional module	Flipped module	
Short term	Mean (score)	31.88	51.4	<0.0001
	Standard deviation	19.95	14.09	
Long term	Mean (score)	52.33	59.0	<0.095
	Standard deviation	22.1	17.2	

**Figure 3:**
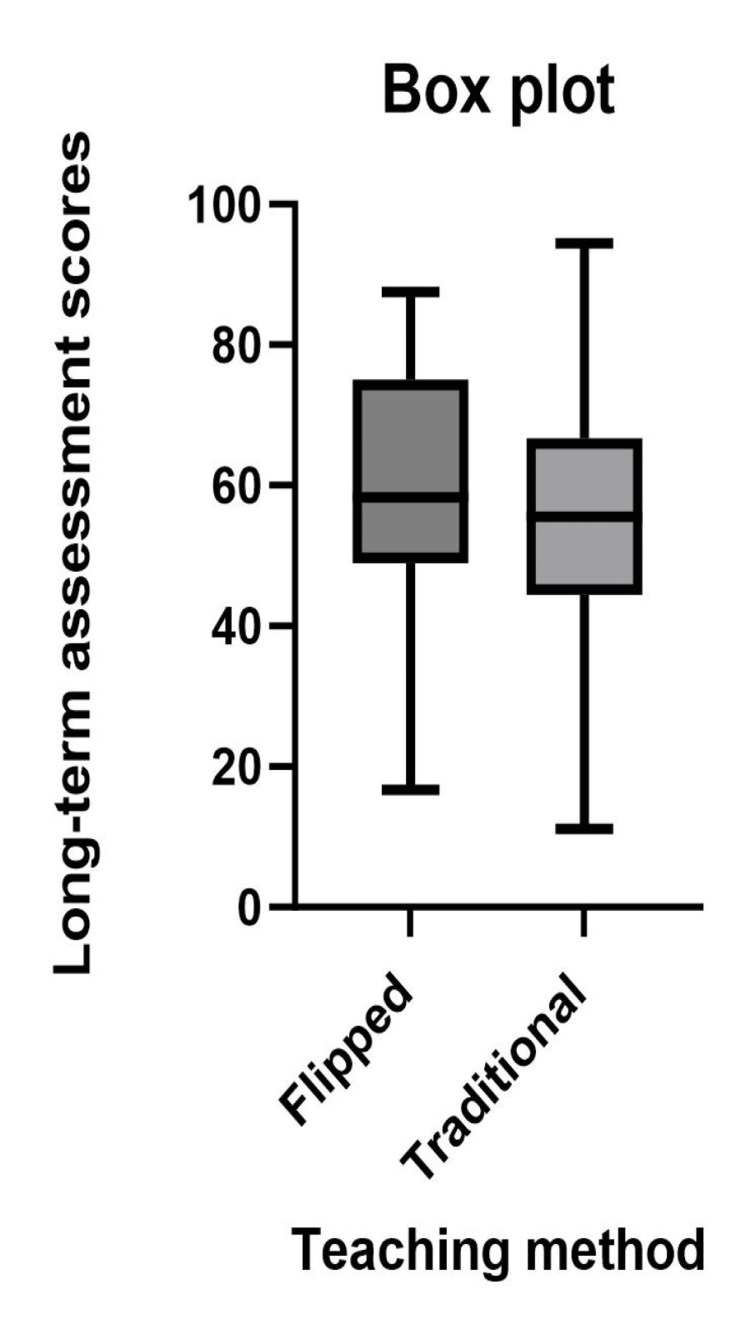
Comparison of long-term assessment scores between flipped and traditional method.

The assessment scores for the long term had increased for both teaching modules when compared with the respective assessment scores for the short term. The difference between the scores of short-term and long-term assessments for the traditional module is higher than the difference for the flipped module. The assessment scores of the flipped module are higher compared to the assessment scores of traditional modules in the short term as well as the long term (Table 1 and Figures 2-3). A positive difference was observed in the comparison of the long-term assessment scores between flipped and traditional modules, with a p-value of <0.095 (Figure 3).

The assessment scores for the long term were found to be uniformly elevated for all the students in the flipped module. A higher number of students gained more than 50% scores in short-term assessment for the flipped module compared to traditional modules. Few students gained less than 35% scores in short-term assessment for the flipped module when compared with traditional modules (Table 2).

**Table 2:** Count of students based on the percentage scores for flipped and traditional modules.

		Percentage scores					
		More than 50%		Less than 50%		Less than 35%	
		Traditional	Flipped	Traditional	Flipped	Traditional	Flipped
No. of students (out of 50)	Short term	8	40	42	10	31	6
	Long term	28	33	22	17	9	6

**Figure 4:**
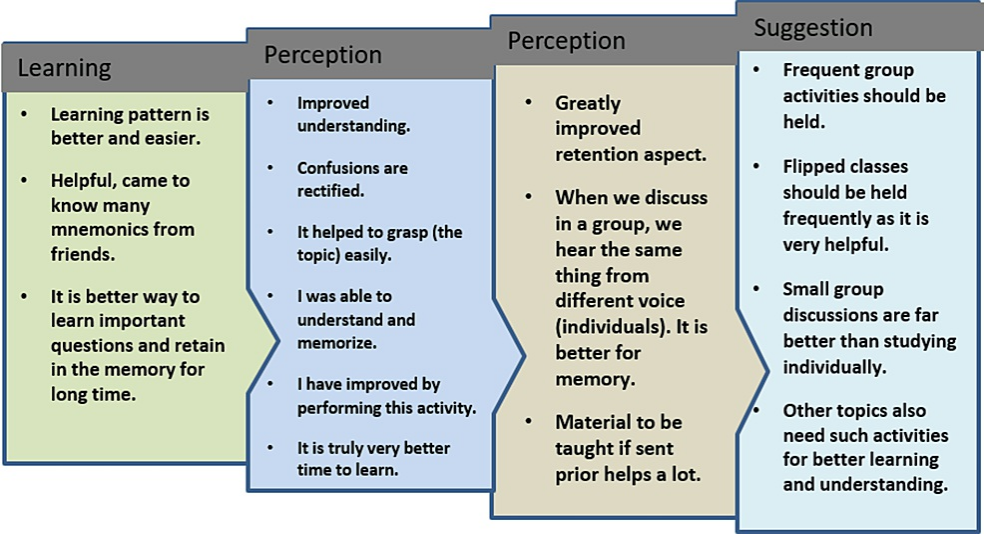
Thematic analysis of the students’ feedback.

A comparison between the short-term and long-term assessment scores of students gaining < or >50% and 35% marks in the short-term assessments for both modules is presented in Table 3. As the same set of students were exposed to both teaching methods on different occasions, themes from the comparative feedback obtained have been noted in Figure 4.

**Table 3:** Comparison between short- and long-term assessment scores among students gaining < or > 50% and 35% marks in their short-term assessments for both modules.

Teaching method	Basis of analysis (marks)	Mean of differences	Standard deviation of differences	Standard error of mean of differences	P-value	Statistical significance
Flipped	>50%	4.45	15.91	2.516	0.0848	No
	<50%	21	15.8	4.997	0.0023	Yes
	>35%	6.87	16.84	2.483	0.0082	Yes
	<35%	18	19.17	9.584	0.157	No
Traditional	>50%	11.49	14.03	3.218	0.0022	Yes
	<50%	27.53	21.28	3.822	<0.0001	Yes
	>35%	12.6	16.57	3.314	0.0009	Yes
	<35%	30.26	20.08	4.016	<0.0001	Yes

## Discussion

In terms of assessment of learning outcomes, researchers have demonstrated higher exam grades for students using a flipped classroom approach as compared to students learning through traditional methods. The improvement in the assessment scores explains the success of flipped classroom teaching techniques. This is consistent with the previous studies [7].

The impact on the generation effect and the testing effect pertaining to this study has been observed. The generation effect is the ability to regenerate knowledge at a higher rate when it is self-generated compared with read or experimenter-provided information [8]. The generation effect is more flipped than the traditional method, as is evident from the difference in the short-term (statistically significant) and long-term assessment scores (Table 1).

The testing effect studies the positive effect of information retrieval on long-term memory retention [8]. This has improved in both methods. The long-term assessment scores for the flipped module are relatively higher than for traditional modules, though statistically not significant.

It is substantiated by students that after having gone through the study material provided prior, when they discuss it in a group and hear the same from fellow colleagues, it helps them to remember and retain it for a long time. This finding is in accordance with Ebbinghaus’ Forgetting Curve, which states that there is a direct correlation between memory and time [9]. Generally, forgetting is a rapid process. But to remember things’, repeated practice is required. Experiments have shown that if one memorises things repeatedly within an hour, one will remember them for a day. If one memorises one day later, one will remember for one week [9].

Students like flipped classroom teaching and prefer it over learning alone. Students take responsibility and control of their own learning in terms of the pace of study, mastery of content, and coming to class prepared. Each student has his/her own method of learning, and during flipped sessions, they interact and become aware of new techniques of learning for the same topic, like mnemonics, sharing personal experiences, etc. This is beneficial in building concepts and in the development of critical thinking. Also, they are more receptive and do not hesitate to clear their doubts during such sessions.

While actively participating in the discussions during the flipped classroom, the students can reach higher levels in Bloom’s taxonomy, such as application, analysis, and synthesis of knowledge, and attain leadership qualities and the ability to work in a team (Figure 1). This is in conformity with the previous reports, which compare three different instructional strategies in an information systems spreadsheet course. The study showed that students attending the flipped classroom course were more satisfied with the learning environment compared to the other groups [4].

Students expressed that the flipped classroom is very useful and that it should be used more often. This is in line with the previous study, where analysis of pharmacy students’ experiences of flipped classroom courses revealed that students who learned through a flipped classroom approach considered themselves more engaged than students attending traditional courses [10].

According to one of the studies performed on the graduates in the internal medicine department, residents interviewed expressed significant concerns about the time and motivation to watch the required videos outside of scheduled teaching sessions [11]. As the first-year medical students have no additional clinical responsibilities like the internal medicine residents, they did find time to go through the pre-course material. When the students find it difficult to put in the required preparatory time and effort for viewing the pre-course material, in that case, they will join the flipped classroom passively [12]. Thus, the limitation of the study was ensuring whether students coming for the flipped classroom sessions were going through the material beforehand or joining the discussion passively.

We suggest that along with the study material, we also give a list of questions pertaining to the topic. At the start of the flipped classroom, a quiz in the form of objective questions of higher order, pictorial questions, word searches, crossword puzzles, etc. can be held, which will act as a major factor in increasing the students’ interest in grasping the content to be discussed. The instructor should identify any sort of misconception related to the topics from the pre-class material. Based on the concepts gained, the instructor can provide remedial actions, if required, in the form of reviewing specific aspects of the material again [13].

The difference between the scores of long-term and short-term assessments for the traditional module was higher than the similar difference between the scores of long-term and short-term assessments for the flipped module in the study. Also, the comparison between the short-term and long-term assessment scores of students gaining < or >50% and 35% marks in the short-term assessments for the traditional module has significant results (Table 3). For the flipped module, only two comparisons are significant (Table 3).

A possible reason for this difference is that the students gained higher short-term assessment scores after the flipped sessions and very low short-term assessment scores for the traditional module. So, the difference between the short- and long-term assessment scores for the students is less for the flipped module. It can also be explained by the fact that students tend to thoroughly read the confined syllabus just before the formative assessment examinations, so the difference in the long-term assessment scores is statistically insignificant. Also, the students underwent routine dissection sessions after short-term assessments for both modules. This is likely to impact the rise in scores, which is applicable to both teaching methods.

This can also be explained by the different experiential learning styles of individuals. Kolb’s learning style inventory describes four learning styles: divergent (concrete experience and reflective observation), assimilative (abstract conceptualization and reflective observation), convergent (abstract conceptualization and active experimentation), and accommodative (concrete experience and active experimentation). These learning styles have been found to affect study duration and theoretical anatomy course scores [14].

In our study, the finding of a higher number of students obtaining higher scores through the flipped classroom method in both short-term and long-term assessments (Table 2) suggests tangible benefits attainable through this method. This is a unique finding for our study (Tables 1-2).

However, if the students fail to revise the portion covered in flipped or traditional classes before the formative or summative assessments, then it is likely that their scores may decrease. The ability of each individual student to comprehend the topic thoroughly will also determine their assessment scores.

The flipped classroom module emphasizes more on understanding the concepts than on writing theoretical answers, which are necessarily present in the design of the formative assessments. The students may face difficulty in writing theoretical answers based on the flipped classroom module. This could be a confounding factor. Hence, we suggest a surprise assessment for these topics covered in the flipped classroom. This will also give a fair idea about the actual impact of these sessions on long-term retention. When such surprise assessments for the flipped module are designed to engage the students in answering theoretical questions, they will outperform in the final test as compared to those who study only for the formative assessments [8].

In the traditional teaching method, the focus is on content delivery and not on individual needs. This restricts the clarification of concepts and doubts for each student. However, in the flipped classroom method, individualised, differentiated learning was facilitated by personalised assignments. So, there is a need for more facilitators during the flipped session. The time and effort required from the faculties as facilitators will be a challenge in the initial period. However, this exercise, once done, will be very effective and time-saving in the long run [4].

If the entire course material can be appropriately converted into flipped sessions, it will serve as a time saver for the students who do not need in-class help. Students can focus their efforts on their individual learning needs. Thus, they are not left behind by class discussions that go too fast or become bored by the class time that is spent covering content they already know. Students need to attend class only if they need help beyond what is provided by other learning resources. This can be helpful for efficient students to cover the course material in a shorter period of time and use the additional time to gain psychomotor skills pertaining to patient care [4].

The flipped classroom can also be converted into e-flipped classroom sessions. We suggest the usage of learning management systems for all subjects, which will have pre-recorded videos, PPTs, notes, etc., so the students will not waste time surfing the internet to understand concepts and clear doubts. This authentic study material can be customised based on the local needs of the institution.

## Conclusions

The active participation of students, sharing of their fundamental knowledge, and style of learning during flipped classroom sessions contribute to the effective learning of students. Flipped classroom sessions can be incorporated frequently for different topics and disciplines. It can be modified to be used for online teaching sessions and assessments. Thus, the study concludes that the flipped classroom method serves as an advantageous tool and motivating factor for effective learning, understanding, and retention of conceptual and factual content.
